# Understanding flare in axial spondyloarthritis: novel insights from daily self-reported flare experience

**DOI:** 10.1093/rap/rkab082

**Published:** 2021-11-15

**Authors:** Rosemarie Barnett, Stanley Ng, Raj Sengupta

**Affiliations:** 1 Rheumatology, Royal National Hospital for Rheumatic Diseases, Royal United Hospitals NHS Foundation Trust; 2 Department for Health, University of Bath, Bath; 3 White Swan Charity, Exeter; 4 Department of Pharmacy and Pharmacology, University of Bath, Bath, UK

**Keywords:** axial spondyloarthritis, AS, flare, symptoms, remote monitoring, patient-reported outcomes

## Abstract

**Objectives:**

Our objective was to explore daily self-reported experiences of axial SpA (axSpA) flare based on data entered into the Project Nightingale smartphone app (www.projectnightingale.org), between 5 April 2018 and 1 April 2020.

**Methods:**

Paired *t*-tests were conducted for mean_flare_on and mean_flare_off scores for each recorded variable. The mean estimated difference between flare and non-flare values for each variable was calculated with 95% CIs. Mean, S.d. and range were reported for flare duration and frequency. Participants with ≥10 days of data entry were included for affinity propagation cluster analysis. Baseline characteristics and mean flare on *vs* mean flare off values were reported for each cluster. Welch’s *t*-test was used to assess differences between clusters.

**Results:**

A total of 143/189 (75.7%) participants recorded at least one flare. Each flare lasted a mean of 4.30 days (S.d. 6.82, range 1–78), with a mean frequency of once every 35.32 days (S.d. 65.73, range 1–677). Significant relationships were identified between flare status and variable scores. Two clusters of participants were identified with distinct flare profiles. Group 1 experienced less severe worsening of symptoms during flare in comparison to group 2 (*P* < 0.01). However, they experienced significantly longer flare duration (7.2 *vs* 3.5 days; *P* < 0.01), perhaps indicating a prolonged, yet less intense flare experience. Groups were similar in terms of flare frequency and clinical characteristics.

**Conclusions:**

Two clusters of participants were identified with distinct flare experiences but similar baseline clinical characteristics. Smartphone technologies capture subtle changes in disease experience not currently considered in clinical practice.

Rheumatology key messages

- Daily self-reported smartphone data identified two distinct clusters of people living with axSpA who had different flare experiences.
- Despite differences in flare duration and symptoms, baseline clinical measures were similar between clusters.
- Smartphone technologies capture subtle changes in disease experience not currently considered in clinical practice.

## Introduction

Axial SpA (axSpA) is a chronic, inflammatory disease characterized by alternating periods of flare and more stable disease activity. Flares are often both unpredictable and debilitating and a greater understanding of their nature and outcome is therefore important to both those living with axSpA and clinicians [1, 2]. Over the last decade, although rapid advances have been made in terms of our understanding of axSpA, the natural history of the disease remains elusive. It has been hypothesized that the presence of early, severe disease flares (often associated with a worsening of symptoms or increased disease activity) may allow for early identification of people living with axSpA who may develop more severe disease [3–6]. Indeed, severe flare has been identified as a poor prognostic factor in axSpA, particularly in early disease [4]. It has therefore been suggested that early, aggressive treatment of severe flares in axSpA may improve long-term outcomes.

Despite the frequent use of the concept ‘flare’ within rheumatic condition terminology, an accepted, consistent definition of a flare does not yet exist for axSpA. In recent years there have been attempts to define flare in both axSpA and other chronic rheumatic conditions such as RA based on validated composite indices or through qualitative retrospective investigation of flare states [3, 7–9]. Indeed, there is growing interest in the concept of flare and in characterizing the lived experiences behind this multidimensional phenomenon [1, 6, 7, 9–17]. Such an understanding is critical to better characterize the natural history of the condition and in the future may facilitate optimization/personalization of available treatments. The problematic nature of defining a flare in axSpA lies in part in the multifaceted, heterogeneous nature of the disease. This problem was clearly demonstrated by Gossec *et al.* [7], whereby, in a preliminary attempt to classify flare, 27 different flare definitions were identified among 38 publications on axSpA.

In prior studies investigating flare experiences, those living with axSpA have often been asked to recall a history of flare or prior experience of flare [3–6]. However, this retrospective characterization is subject to recall bias and may not provide an accurate picture of the lived day-to-day reality. Recently introduced smartphone technologies for the daily monitoring of disease symptoms and activity provide unique insights into the daily experiences of individuals with chronic, fluctuating conditions [18–25]. Such technologies may allow for a more accurate investigation of flare experiences [12, 16, 17].

In the present study we conducted an exploratory analysis on a dataset of participants entering daily symptoms and behaviour into the Project Nightingale (uMotif) app (www.projectnightingale.org). Our objective was to explore individual’s self-reported experiences of flare. We hoped to characterize the constituents of flare, the frequency and duration of flare and whether people living with axSpA could be clustered based on their similar experiences. We then attempted to further characterize these clusters of participants to provide detailed insights into potential distinct subtypes of flare experience.

## Methods

### Overview of Project Nightingale

Since April 2018, people living with axSpA under the care of the Royal National Hospital for Rheumatic Diseases (RNHRD), Royal United Hospitals NHS Foundation Trust (RUH), Bath, UK, have been eligible to participate in Project Nightingale. Project Nightingale was created to allow people living with axSpA to track daily symptoms and behaviour via their smartphone device to gain further insights into the nature of their condition.

All participants are invited to track 10 variables via the uMotif smartphone app, including 8 fixed and 2 optional variables. Fixed variables are tracked by all participants and include pain, mood, fatigue, sleep, stress, flare, recommended exercise and anti-inflammatory use. Two optional variables are chosen by each participant from the following: caffeine intake, hot flushes, adherence to medication, screen time, confidence in self-management, eyesight, hydration, chest pain, flare of psoriasis, impact of menstrual cycle, red painful eyes, smoking habits and blood in stool. The variables and associated scales were designed by the lead consultant for axSpA at the RNHRD to optimize clinical relevance, following years of regular, detailed and empathetic interaction with people living with axSpA.

Participants are asked how they are feeling each day via the app. They rate each variable on a 5-point Likert scale. The interface for recording each outcome is displayed as a flower-like visualization whereby each petal represents one of the 10 tracking variables (Supplementary Fig. S1, available at *Rheumatology Advances in Practice* online). Participants are required to drag their finger from the centre of the flower to the outer edge of each petal to record their symptoms. For each variable, a score of 1 equates to the less healthy or desirable outcome, whereas a score of 5 represents the most healthy/positive outcome or behaviour. For example, for pain, 1 = debilitating pain and 5 = no pain. The flower-motif recording interface acts as a visual metaphor, whereby a full flower represents the most healthy or optimal outcomes. Participants receive daily reminders for data entry as a notification to their smartphone if data has not already been entered. In the uMotif app settings, participants can choose to opt out of reminders or alter their time and frequency.

### Data collection

For the present study we utilized smartphone data collected via the Project Nightingale (uMotif) app between 5 April 2018 and 1 April 2020. The South West–Central Bristol UK local research ethics committee for National Health Service research approved the study and all patients provided written informed consent (Bath Spondyloarthritis Biobank; REC reference 13/SW/0096). Clinical data were collected based on routine assessment at the RNHRD. Baseline measures were extracted at the visit date closest to Project Nightingale registration, restricted to visit dates within 90 days of Project Nightingale registration. Data from participants’ wearable and smart device applications were downloaded regularly and incorporated into the patient record.

### Statistical methods

For participants with at least one flare and non-flare set of recorded variables, data were aggregated to one row per participant, containing mean values with and without flare for each petal variable. For example, Participant 1 would have an average_pain_flare_on feature and an average_pain_flare_off feature for each variable. Paired *t*-tests were conducted for each variable to investigate which variables correlated with flare status. The difference between the flare_on and flare_off features were taken for each pair to create a set of ‘difference’ features to capture the effect of a flare on each petal variable for each participant. The mean estimated difference between flare and non-flare values for each variable was calculated with its 95% CI. The mean, S.d. and range were reported for flare duration and flare frequency. For the flare duration calculation, two logged periods of flare occurring within 3 days were considered as one period of flare if missing 1 day of data between entries.

For the cluster analysis, Project Nightingale participants with <10 days of data entry were excluded. Difference features for each variable for each participant were normalized to between −1 and 1 and then used for clustering. Affinity propagation was used as the clustering algorithm via the apcluster R package (R Foundation for Statistical Computing, Vienna, Austria) [26]. negDistMat (*r* = 2) was used for the similarity matrix, squaring the distance measures between participants to calculate similarities [27]. *q* = 0 was used to minimize the number of clusters found. Given the size of the dataset (129 participants), it was decided to reduce the number of clusters in order to achieve a meaningful sample size for each cluster [28].

Baseline characteristics and mean flare on *vs* mean flare off values were reported for each cluster. Welch’s *t*-test was used to assess differences between clusters.

### Patient and public involvement (PPI) statement

Project Nightingale was established through a strong collaboration between the RNHRD (RUH, Bath) and consultant R.S., engagement with relevant stakeholders [people living with axSpA and healthcare professionals (HCPs)], the charity White Swan and the Bath Institute for Rheumatic Diseases (BIRD). This has facilitated PPI from project initiation. Petal tracking variables were determined by R.S. based on decades of clinical experience and interactions with people living with axSpA. Additional optional variables were also added to the scope based on patient feedback at Project Nightingale information days. These regular Project Nightingale and axSpA information days organized by BIRD have facilitated patient–HCP–researcher discussion, knowledge exchange, participant feedback and dissemination of results. Such interactions and collaborations have informed advancement of future Project Nightingale research plans and app innovations.

PPI has been maintained during the coronavirus disease 2019 pandemic via regular Project Nightingale patient–HCP–researcher discussions during the well-established RNHRD axSpA rehabilitation course. A Project Nightingale BIRD podcast episode and Facebook Live event with the National Axial Spondyloarthritis Society have also facilitated PPI. The Project Nightingale blog and Twitter have facilitated regular research updates and dissemination of results to the wider axSpA community. This has allowed for further patient participation and discussion of experiences [29, 30].

## Results

Between 5 April 2018 and 1 April 2020, 189 patients consented for research and logged a mean of 156.78 days of data (S.d. 199.60, range 1–711). A total of 143/189 (75.7%) participants recorded at least 1 flare, with 1349 flares recorded in total. Each flare lasted a mean of 4.30 days (S.d. 6.82, range 1–78), with a mean frequency per participant of once every 35.32 days (S.d. 65.73, range 1–677). Significant relationships were identified between flare status and variable scores (Table 1). Small but significant (*P* < 0.01) estimated differences were found between flare and non-flare scores for pain, fatigue, sleep quality, exercise, mood, anti-inflammatory use, stress, confidence in self-management and chest pain.

**Table 1:** Paired *t*-tests: flare *vs* non-flare scores for each variable

Estimated difference^a^	*P*-value	95% CI (lower limit)	95% CI (upper limit)	*n*	Variable
−0.788^*^	0.000	−0.898	−0.679	143	Pain
−0.599^*^	0.000	−0.706	−0.491	143	Fatigue
−0.228^*^	0.000	−0.303	−0.153	142	Sleep quality
−0.296^*^	0.000	−0.416	−0.177	143	Recommended exercise
−0.381^*^	0.000	−0.463	−0.298	143	Mood
0.140^*^	0.000	0.090	0.191	143	Anti-inflammatory use
−0.343^*^	0.000	−0.459	−0.228	143	Stress
−0.038	0.348	−0.119	0.043	59	Caffeine intake
−0.404	0.012	−0.709	−0.100	19	Hot flushes
−0.018	0.685	−0.106	0.071	26	Adherence
−0.289	0.092	−0.630	0.053	18	Screen time
−0.500^*^	0.000	−0.693	−0.307	36	Confidence in self-management
−0.123	0.121	−0.281	0.035	24	Eyesight
−0.136	0.123	−0.311	0.038	51	Hydration
−0.419^*^	0.006	−0.707	−0.131	28	Chest pain
−0.024	0.681	−0.238	0.191	3	Flare of psoriasis
−0.310	0.175	−0.781	0.161	13	Menstrual cycle
0.101	0.599	−0.345	0.547	7	Red painful eyes
0.132	0.704	−1.163	1.427	3	Smoking today
0.224	0.371	−1.655	2.104	2	Blood in stool

*n* = number of patients with both a flare and non-flare entry for each variable. Higher variable scores indicate more positive outcomes (e.g. a higher pain score indicates less pain).

a Estimated difference between flare and non-flare entries [e.g. on average, the mean pain score of a flare entry is 0.67 (CI 0.56, 0.78) less than a non-flare entry].

* *P* < 0.01.

**Fig. 1:**
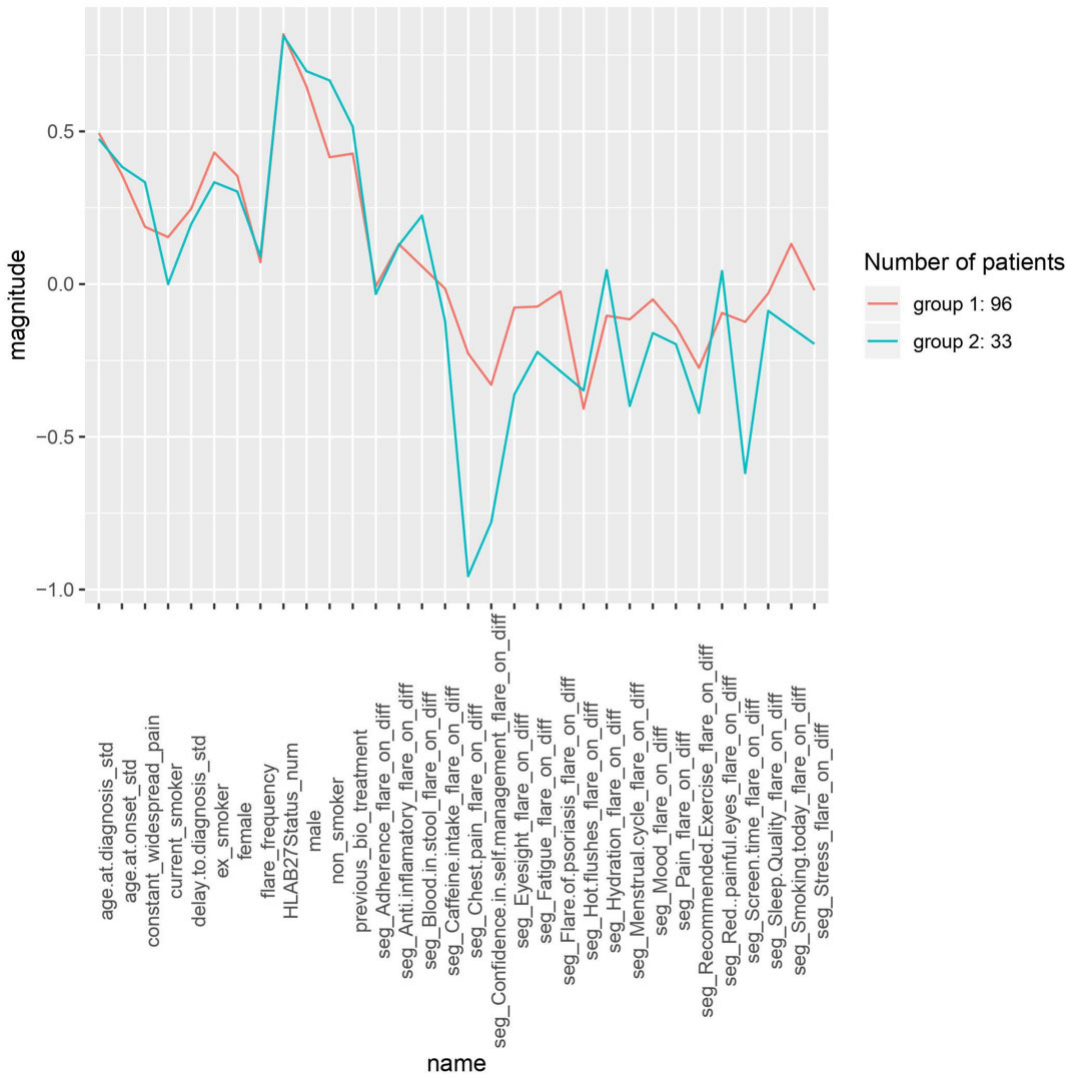
Differences (normalised to [−1, 1]) in petal values between two clusters of patients recording self-reported flare in the Project Nightingale (uMotif) app

Between 5 April 2018 and 1 April 2020, 129 patients had registered for participation in Project Nightingale and provided ≥10 days of data entry suitable for the cluster analysis. Two clusters of participants were identified based on distinct profiles of uMotif petal symptom scores during flares, using non-flare scores as a baseline comparator (Fig. 1, Table 2). Group 1 appeared to experience less severe worsening of pain, fatigue, sleep, mood and stress during flare (*vs* non-flare) compared with group 2 (*P* < 0.01). However, this group also experienced significantly longer flare duration (7.2 *vs* 3.5 days; *P* < 0.01) (Supplementary Table S1, available at *Rheumatology Advances in Practice* online), perhaps indicating a more prolonged, yet less intense flare experience. Although not reaching significance due to small sample size, group 2 also demonstrated a greater decrease (worsening) in the score for chest pain, confidence in self-management, eyesight, flare of psoriasis, impact of menstrual cycle and screen time. Changes in anti-inflammatory use and recommended exercise during flare *vs* non-flare appeared similar between the two groups, perhaps suggesting similar behaviours while attempting to resolve flares.

**Table 2:** Absolute differences in petal values between flare and no flare values for two clusters of patients recording self-reported flare in the Project Nightingale (uMotif) app

Petal Variable	Group 1 (*N* = 96)		Group 2 (*N* = 33)		*P*-value
	Difference^a^	*n*	Difference^a^	*n*	
Pain_flare_on_diff	−0.694	96	−0.984	33	0.007
Fatigue_flare_on_diff	−0.368	96	−1.111	33	0.000
Sleep.Quality_flare_on_diff	−0.151	96	−0.438	33	0.000
Recommended.Exercise_flare_on_diff	−0.274	96	−0.422	33	0.300
Mood_flare_on_diff	−0.251	96	−0.800	33	0.000
Anti.inflamatory_flare_on_diff	0.131	96	0.128	33	0.960
Stress_flare_on_diff	−0.101	96	−0.980	33	0.000
Caffeine.intake_flare_on_diff	−0.015	43	−0.122	11	NA
Hot.flushes_flare_on_diff	−0.408	17	−0.348	1	NA
Adherence_flare_on_diff	−0.008	13	−0.032	11	NA
Screen.time_flare_on_diff	−0.124	12	−0.619	6	NA
Confidence.in.self.management_flare_on_diff	−0.330	23	−0.779	9	NA
Eyesight_flare_on_diff	−0.077	18	−0.362	4	NA
Hydration_flare_on_diff	−0.103	32	0.046	14	NA
Chest.pain_flare_on_diff	−0.227	19	−0.956	4	NA
Flare.of.psoriasis_flare_on_diff	−0.024	3	NA	0	NA
Menstrual.cycle_flare_on_diff	−0.115	8	−0.399	2	NA
Red.painful.eyes_flare_on_diff	−0.094	4	0.043	2	NA
Smoking.today_flare_on_diff	0.132	3	NA	0	NA
Blood.in.stool_flare_on_diff	NA	0	0.224	2	NA

a Absolute difference from baseline (non-flare).

NA, sample size too small to determine statistical significance.

Group 2 reported slightly (petal score difference <0.5) better sleep quality (*P* = 0.022) and very slightly higher levels of recommended exercise (*P* = 0.026) than group 1 when not in flare, despite worse scores for pain (*P* = 0.043), fatigue (*P* = 0.001), mood (*P* = 0.031) and stress (*P* < 0.001) during flare (Table 3). No significant differences were found between groups for pain, fatigue, mood, anti-inflammatory use or stress when not in flare.

**Table 3:** Mean flare on and flare off values for two clusters of patients recording self-reported flare in the Project Nightingale (uMotif) app

Petal variable	Group 1 (*N* = 96)		Group 2 (*N* = 33)		*P*-value
	Mean	S.d.	Mean	S.d.	
Flare on					
Pain	3.083	0.638	2.850	0.529	0.043
Fatigue	3.077	0.967	2.555	0.614	0.001
Sleep quality	3.177	0.594	3.192	0.668	0.910
Recommended exercise	3.219	1.152	3.513	0.994	0.166
Mood	3.051	0.799	2.693	0.801	0.031
Anti-inflammatory use	0.552	0.408	0.575	0.373	0.770
Stress	3.875	0.720	3.070	0.902	0.000
Flare off					
Pain	3.777	0.581	3.834	0.590	0.635
Fatigue	3.445	0.805	3.666	0.657	0.122
Sleep quality	3.328	0.545	3.630	0.657	0.022
Recommended exercise	3.494	1.019	3.934	0.934	0.026
Mood	3.302	0.740	3.494	0.708	0.191
Anti-inflammatory use	0.421	0.402	0.447	0.398	0.753
Stress	3.977	0.657	4.049	0.716	0.609

The baseline (at Project Nightingale registration) characteristics of participants in each cluster group are presented in Table 4. Both groups were similar in terms of gender, HLA-B27 status and other clinical characteristics such as spinal mobility (BASMI). However, group 1 had a significantly greater proportion of smokers (*P* < 0.001) and group 2 had a significantly greater proportion of people who had never smoked (*P* < 0.05).

**Table 4:** Baseline^a^ characteristics of two clusters of patients recording self-reported flare in the Project Nightingale (uMotif) app

	Group 1 (*N* = 96)			Group 2 (*N* = 33)			*P*-value
Baseline^a^ values	Mean	S.d.	*n*	Mean	S.d.	*n*	
BASMI score, mean (S.d.)	3.173	2.069	59	3.533	2.082	18	NA
BASDAI score, mean (S.d.)	3.680	1.733	70	3.852	2.081	21	0.732
BASFI score, mean (S.d.)	3.627	2.568	66	3.715	2.214	20	0.882
EQ-5D, mean (S.d.)	0.618	0.191	53	0.659	0.240	20	0.499
Pain and discomfort	2.585	0.663	53	2.400	0.940	20	0.427
Anxiety and depression	1.906	1.005	53	1.600	0.754	20	0.167
Patient global, mean (S.d.)	4.018	2.057	57	3.429	2.420	21	0.329
ASQoL, mean (S.d.)	8.060	4.716	59	7.935	5.234	21	0.924
Work productivity impairment, mean (S.d.)	3.640	2.691	25	4.000	3.162	13	NA
Activity impairment, mean (S.d.)	4.240	2.722	50	3.950	2.964	20	0.708
Proportion of employed	0.592	0.497	49	0.650	0.489	20	0.658
Proportion of females	0.354	0.481	96	0.303	0.467	33	0.592
Proportion of males	0.646	0.481	96	0.697	0.467	33	0.592
Proportion HLA-B27 positive	0.818	0.388	88	0.813	0.397	32	0.945
Proportion of current smokers	0.154	0.364	65	0.000	0.000	21	0.001
Proportion of ex-smokers	0.431	0.499	65	0.333	0.483	21	0.431
Proportion of non-smokers (never smoked)	0.415	0.497	65	0.667	0.483	21	0.047
Proportion ever treated with bDMARDs	0.427	0.497	96	0.515	0.508	33	0.391
Proportion with CWP	0.188	0.392	96	0.333	0.479	33	0.121

a At visit date closest to Project Nightingale registration; restricted to visit dates within 90 days of Project Nightingale registration date.

CWP, chronic widespread pain.

## Discussion

To our knowledge, this is the first study to investigate, characterize and group daily self-reported flare profiles in people with axSpA utilizing a smartphone application and remote data collection. Two distinct clusters of participants were identified. One group reported significantly shorter flare duration (*P* < 0.01), however, they experienced a significantly greater worsening of pain, fatigue, mood, sleep and stress during flare (*P* < 0.01), perhaps indicating a shorter, although more intense, flare experience. The number and frequency of flares were similar between clusters, as were baseline clinical measures such as BASMI, BASDAI, BASFI and quality of life [measured through the Ankylosing Spondylitis Quality of Life (ASQoL) questionnaire]. Smartphone technologies therefore have the potential to capture subtle, potentially critical changes in disease activity that are not currently considered in clinical practice. Although the long-term significance of these is yet to be explored, such work is planned in our future research agenda. Furthermore, the study of such daily self-report data may in the future allow for prediction of flare based on patterns of symptoms/behaviour or enable a greater understanding of behaviours that lead to earlier resolution of flare. This may facilitate earlier targeting and prevention of flares to reduce flare frequency and duration, to ultimately improve the quality of life for patients.

Prior qualitative work by Brophy and Calin in 2002 [3] also identified two types of flare, localized and generalized, based on group discussions with 214 patients over the period of 1 year. All participants had experienced a localized flare involving pain and immobility in one area, sometimes accompanied by fatigue and emotional symptoms. In contrast, only 40% (85/214) of participants had experienced generalized flares, involving the whole body. This was described as an infrequent event whereby all symptoms were experienced to the extreme. Individuals reporting generalized flares described the localized flares as not a ‘true’ flare—perceiving localized increases in disease activity as incomparable to the crippling, acute and devastating phenomenon of a whole-body flare. Similar experiences of localized (minor) or generalized (major) flares have been characterized in later studies by Stone *et al.* in 2008 [6] and a follow-up study in 2010 [5]. In the present study we were unable to determine the location of flares. However, our results appear broadly consistent in terms of one group of patients experiencing more intense, debilitating flares, with greater changes in symptoms such as pain, fatigue, mood, sleep and stress. In the present study, this group experiencing more severe flares again appeared to involve the minority of participants [26% of participants in the present study (33/129)].

Our average flare duration may appear less than previously reported. In 2002, Brophy and Calin [3] described the majority of flares as short-term (days to weeks), broadly in agreement with the present study. However, in 2010, Cooksey *et al.* [5] reported a mean flare duration of 2.4 weeks, compared with an average duration of 7.2 and 3.5 days for group 1 and group 2, respectively, in the present study. This is likely because our flare duration calculation was quite strict, in that a flare required subsequent days of uMotif flare entries to be considered as ‘continued’. Just 1 day of missing data was permitted. For example, if a participant recorded a flare on a Monday and Wednesday but with missing data on Tuesday, this would be recorded as a single period of flare. However, if a participant recorded a flare on a Monday and Thursday with 2 missing days of data, this would be considered as two separate periods of flare. This was defined in alignment with a more recent study by Jacquemin *et al.* [14], whereby the majority of reported flares lasted ≤3 days. However, this definition may have considerably underestimated the flare duration in the present study. The past 10–20 years have shown dramatic advances in our understanding of axSpA, including the introduction of the widespread use of biologics, improved treatment strategies and a change in definition of disease (to include non-radiographic axSpA in addition to AS). Therefore this may have contributed to the differences in flare duration seen in the present study and the study by Jacquemin *et al.* [14]. Indeed, both earlier studies (Brophy and Calin [3], Cooksey *et al.* [5]) included only people with AS, but not non-radiographic axSpA, perhaps further contributing to the disparity in flare duration.

It is also important to note that despite short flare duration in the present study, the mean flare frequency per participant was once every 35.32 days (S.d. 65.73). This suggests that there is still a need for optimization/personalization of treatments in axSpA in order to reduce the frequency of debilitating flare and potential associated poor clinical outcomes and work impairment [4, 6, 31, 32].

Beyond the importance of flare characterization in clinical practice, flare also represents an important endpoint to consider in clinical trials. As a potential indicator of disease severity, flare assessment is vital to understanding disease status or treatment efficacy and is of particular importance in tapering or discontinuation trials [33–36]. There has recently been an attempt to quantify a single definition of flare based on validated composite indices for the purpose of harmonizing trial designs in axSpA [7, 9]. However, it is important to distinguish between the necessarily stricter, arbitrarily homogeneous definition of flare that is required in clinical trials *vs* the highly variable, highly individualized flare experiences of those living with axSpA. In clinical practice, in order to move towards optimization and personalization of treatments, the latter definition as explored in the present study may arguably be of greater significance. This may be supported by the fact that in the present study, although group 2 reported significantly worse flare experiences via the uMotif app, we found no significant differences in baseline spinal mobility, disease activity or function as measured by validated BASMI, BASDAI and BASFI measures between the two groups, highlighting the power of smartphone technologies to capture potentially critical fluctuations in disease severity that are too subtle to be observed by traditional, infrequent measurement of existing validated indices. Indeed, future integration of daily self-reported health data into the electronic health record may allow for greater optimization and personalization of treatment outcomes through more accurate reporting of disease experiences [37].

A limitation of the present study is with regard to adherence. Upon registration, participants were encouraged to enter data every day. However, they were told that any data entry may be useful, including restarting after inactive periods. Prior qualitative and quantitative evidence suggests that patients with worse disease experiences in axSpA may be more likely to adhere to self-tracking behaviour [38]. Therefore our results may be biased towards those with more severe disease. Similar results have been reported in the literature for other inflammatory, rheumatic conditions such as RA, where it has been suggested that patients may primarily use self-tracking apps in the case of impending flares [39].

Another potential source of bias in the present study is that the RNHRD is a tertiary hospital receiving both local and specialist referrals. Therefore our cohort may be more severely affected by axSpA or less likely to experience a down period between flares. However, both our own data and data from prior studies from the RNHRD suggest that our cohort of patients reflects the full spectrum of axSpA disease [6, 40]. For example, the population included in the present study showed a range of BASDAI scores from 0 to 8.6 and BASMI scores from 0 to 7.8. Disease duration (from age of onset to age at study consent) ranged from 4 years to 68 years. Furthermore, it is now common practice and recommended for general practitioners to refer all suspected axSpA diagnoses to a specialist centre [41].

## Conclusions

The results of the present study yield novel insights into the characterization of flares in axSpA. Significant relationships were identified between a variety of patient-reported symptoms and flare, including variables that, to our knowledge, have not yet been explored in axSpA. Clustering of daily self-reported symptom data has identified two clusters of people with axSpA who have distinct flare profiles. One group appears to experience significantly longer flare duration. However, this group also experiences less dramatic worsening of pain, fatigue, sleep, mood and stress during flare compared with non-flare. Although we observed differences between the two groups in terms of flare experiences, clinical differences in BASMI, BASDAI and BASFI were not identified, highlighting the potential of smartphone technologies to capture subtle, potentially critical changes in disease activity that are not currently considered in clinical practice.

## Acknowledgements

We acknowledge Charlotte Cavill and Mandy Freeth from the RNHRD, RUH, for extraction of baseline clinical measures from the Bath Spondyloarthritis Research Biobank. We acknowledge our collaborators at uMotif, in particular, Steph Meleck, Director of Professional Services, and Bruce Hellman, CEO & Founder. R.B., S.N. and R.S. contributed to the conception, design and planning of the reported work. S.N. conducted the statistical analysis. R.B. drafted the final publication, which was reviewed/revised and approved by S.N. and R.S. for submission. Our collaborators at uMotif, in particular, Steph Meleck and Bruce Hellman, supported the acquisition of data, as did Charlotte Cavill and Many Freeth from the RNHRD, RUH.

*Funding*: This work was supported by UCB, who provided funding for use of the uMotif app. The time of R.B. was funded by the Sir Halley Stewart Trust. The views expressed in this article are those of the authors and not necessarily those of the trust.

*Disclosure statement*: R.S. has received grants/research support from AbbVie, Celgen, Novartis and UCB and honoraria or consultancy fees from AbbVie, Biogen, Novartis, Celgene, Eli Lilly, Chugai, MSD and UCB. R.S. is also on advisory boards for AbbVie, Biogen, Chugai, Eli Lilly, Novartis and UCB. The remaining authors have declared no conflicts of interest.

## Data availability statement

Due to confidentiality agreements, supporting de-identified participant data can only be made available to bona fide researchers subject to approval and implementation of a data sharing agreement, as determined by the RNHRD, RUH, Bath (data custodians). Requests for data will be evaluated on the basis of the research objectives of the intended project and any conflicts of interest involved. Enquiries about the Project Nightingale dataset should be directed to Rosemarie Barnett (rlb60@bath.ac.uk).

## Supplementary data

Supplementary data are available at *Rheumatology Advances in Practice* online.

## Supplementary Material

rkab082_Supplementary_Data
