# Supplementary material for: Understanding flare in axial spondyloarthritis: novel insights from daily self-reported flare experience
Source: Rheumatol Adv Pract. 2021 Nov 15;5(3):rkab082. doi: 10.1093/rap/rkab082 (PMC8678434; doi:10.1093/rap/rkab082)
Supplement: rkab082_Supplementary_Data [file rkab082_supplementary_data.docx]

## Supplementary material

**Supplementary Figure S1.** Screenshots from the Project Nightingale smartphone app - daily completion of the motif.


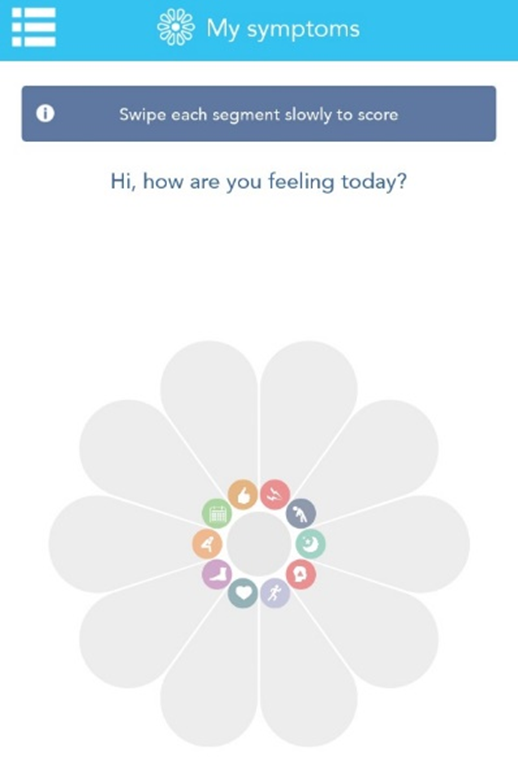

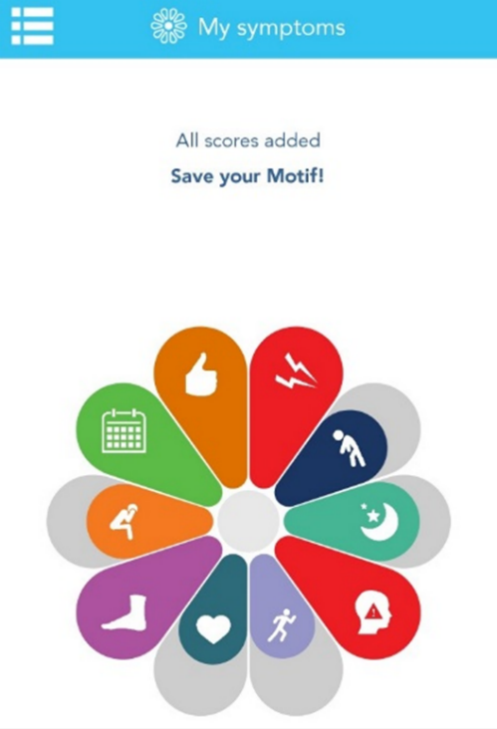


B)

A)

**Supplementary Table S1.** Flare characteristics of two clusters of patients recording self-reported flare in the Project Nightingale (uMotif) app.

| Variable | Group 1  (N=96) | | | Group 2  (N=33) | | | p-value |
| --- | --- | --- | --- | --- | --- | --- | --- |
|  | Mean | SD | N | Mean | SD | N |  |
| *Number of flares* | 10.385 | 12.267 | 96 | 10.091 | 13.689 | 33 | 0.913 |
| *Mean number of active days (days reporting symptoms in the app)* | 223.906 | 210.692 | 96 | 188.818 | 201.750 | 33 | 0.398 |
| *Mean flare frequency^* | 0.072 | 0.069 | 96 | 0.091 | 0.084 | 33 | 0.249 |
| *Mean flare duration (no. of days)* | 7.208 | 10.188 | 96 | 3.530 | 2.756 | 33 | 0.002 |

*^* *Flare frequency reported as a proportion of each participants' number of active days.*
